# The effect of supplementation with vitamins A, B, C, D, and E on disease severity and inflammatory responses in patients with COVID-19: a randomized clinical trial

**DOI:** 10.1186/s13063-021-05795-4

**Published:** 2021-11-14

**Authors:** Mohammad Taghi Beigmohammadi, Sama Bitarafan, Azin Hoseindokht, Alireza Abdollahi, Laya Amoozadeh, Danesh Soltani

**Affiliations:** 1 Anaesthesiology and Intensive Care Department, Imam Khomeini Hospital complex, Tehran University of Medical Sciences, Tehran, Iran; 2 Iranian Center of Neurological Research, Neuroscience Institute, Imam Khomeini Hospital Complex, Tehran University of Medical Sciences, Tehran, Iran; 3 FHMS Clinic, Burnaby Hospital, Neurology Department, University of British Columbia, Vancouver, Canada; 4 Department of Pathology, School of Medicine, Imam Khomeini Hospital Complex, Tehran University of Medical Sciences, Tehran, Iran; 5 Breast Disease Research Center, Tehran University of Medical Sciences, Tehran, Iran

**Keywords:** COVID-19, Vitamin, Supplementation, SOFA, Inflammation, Cytokine

## Abstract

**Background and objective:**

Because of the effect of vitamins on modulating the immune system function, we have evaluated the effect of supplementation with vitamins A, B, C, D, and E in ICU-admitted patients with COVID-19.

**Methods:**

This study was a randomized and single-blinded clinical trial in which 60 subjects were randomly assigned to two groups. The intervention group (*n*=30) received vitamins, and the control group did not receive any vitamin or placebo. The intervention was included 25,000 IU daily of vitamins A, 600,000 IU once during the study of D, 300 IU twice daily of E, 500 mg four times daily of C, and one amp daily of B complex for 7 days. At baseline and after the 7-day intervention, the serum levels of inflammatory markers, vitamins, and the SOFA score were assessed. In addition, the mortality rate and duration of hospitalization were evaluated after the intervention (IRCT registration number: IRCT20200319046819N1/registration date: 2020-04-04, https://www.irct.ir/trial/46838).

**Results:**

Significant changes were detected in serum levels of vitamins (*p* < 0.001 for all vitamins), ESR (*p* < 0.001), CRP (*p* = 0.001), IL6 (*p* = 0.003), TNF-a (*p* = 0.001), and SOFA score (*p* < 0.001) after intervention compared with the control group. The effect of vitamins on the mortality rate was not statistically significant (*p*=0.112). The prolonged hospitalization rate to more than 7 days was significantly lower in the intervention group than the control group (*p*=0.001). Regarding the effect size, there was a significant and inverse association between receiving the intervention and prolonged hospitalization (OR = 0.135, 95% CI 0.038–0.481; *p*=0.002); however, after adjusting for confounders, it was not significant (OR=0.402, 95% CI 0.086–1.883; *p*=0.247).

**Conclusion:**

Supplementation with vitamins A, B, C, D, and E could improve the inflammatory response and decrease the severity of disease in ICU-admitted patients with COVID-19.

## Introduction

COVID-19 is a newly discovered and highly contagious infectious disease with a broad spectrum of clinical manifestations from asymptomatic infection to severe respiratory failure requiring ICU admission [1]. According to available data, about one-fifth of severe cases need to be hospitalized in ICU due to the high possibility of progression to severe complications and death [2, 3]. Therefore, several recent studies have focused on identifying mechanisms and probable therapeutic targets for reducing complications and mortality rates of ICU-admitted patients with COVID-19. It has been shown that, besides the viral load, impaired immune response and subsequent exacerbate inflammatory response to infection are also responsible for increased risk of severe symptoms and mortality in patients with COVID-19 [3–6]. Dietary intake of micronutrients or supplementation with them has well-established beneficial effects on the regulation and integrity of the immune system via the epigenetic modulation of physiological pathways controlling the immune system and the inflammatory process [7, 8]. Current evidence has elucidated that not only, deficiency of vitamins could intensify the COVID-19 disease, but also simultaneous administration of vitamins could synergistically improve the function of the innate and adaptive immune [9, 10]. However, numerous studies have shown the crucial role of vitamin supplementation in modulating the immune system function and improvement of survival in different infectious diseases, some studies have found contradictory results [7, 11, 12].

Given the high mortality rate in severe cases of patients with COVID-19, further studies are needed to make a definite conclusion regarding patients with COVID-19 due to its unpredictable manner. Hence, this study evaluated whether multivitamins can reduce the inflammatory markers, mortality rate, and duration of hospitalization in ICU-admitted patients with COVID-19.

## Methods

### Study design and participants

This study was a randomized and single-blinded clinical trial conducted at Imam Khomeini Hospital to evaluate whether multivitamin supplementation can improve the laboratory and clinical outcomes of ICU-admitted COVID-19 Patients. The Ethics Committee of Tehran University of Medical Sciences approved the study protocol (Ethics number is IR.TUMS.VCR.REC.1399.090). All patients were informed about the goals of the study and signed informed consent. The results of this trial were reported according to the Consolidated Standards of Reporting Trials (CONSORT) guidelines [13]. We included sixty patients according to the below criteria:

#### Inclusion criteria

- Definitive diagnosis (Retrieve RT-PCR Ct values and “25-point severity score” CT scan data from the patients’ medical records) for COVID-19.
- Patients aged between 20 and 60 years,
- Patients who were hospitalized in ICU with severe clinical manifestations of COVID-19, and
- Male and female patients.

#### Exclusion criteria

- Patients who had undergone chemotherapy in one last month,
- Patients who had a history of immunosuppressed diseases such as human immunodeficiency virus (HIV),
- Patients with chronic or acute kidney disease or hepatic dysfunction,
- Patients should not have received any supplements, except for vitamin D, for three months prior to the start of the study (exception for vitamin D was due to the national mandatory program for vitamin D supplementation), and
- Obese or pregnant patients.

The registration code in the clinical trial is IRCT20200319046819N1 and the protocol of this study has been already published (10.1186/s13063-020-04547-0).

### Interventions and randomization

Included subjects were assigned to two groups according to the blocking randomization method based on sex. Thirty patients in the intervention group receive ampules of vitamins for 7 days included; 25,000 IU vitamin A daily, 600,000 IU vitamin D once during the study, 300 IU of vitamin E twice a day, 500 mg vitamin C four times a day, and one ampule daily of B vitamins of Soluvit [thiamine nitrate 3.1 mg, sodium riboflavin phosphate 4.9 mg (corresponding to vitamin B2 3.6 mg), nicotinamide 40 mg, pyridoxine hydrochloride 4.9 mg (corresponding to vitamin B6 4.0 mg), sodium pantothenate 16.5 mg (corresponding to pantothenic acid 15 mg), sodium ascorbate 113 mg (corresponding to vitamin C 100 mg), biotin 60 μg, folic acid 400 μg, and cyanocobalamin 5 μg]. Thirty patients in the control group received no placebo, ICU specialists were aware of the intervention allocation to groups because of critical situation and inaccessibility to similar package for vitamins, and placebo but our participants and statisticians were unaware. Mohammad Taghi Beigmohammadi and Laya Amoozadeh generated the random allocation sequence, enrolled participants, and assigned participants to interventions. The researchers planned to include 30 patients in each group in the present study.

### Outcomes

The main outcomes in this study were the mortality rate, prolonged hospitalization of more than 7 days, the SOFA score, and inflammatory markers that were assessed at baseline and 7th day.

### Clinical and laboratory measurements

Data on demographics, history of the underlying disease, and using respiratory aids for all patients were recorded. Physical examination was performed to assess the vital sign, oxygen saturation, anthropometric variables, and BMI. The SOFA score, known as a tool for evaluating organ failure and the risk of mortality in the ICU, by considering the involvement of 6 organs including respiratory, hepatic, central nervous system, renal, and coagulation system, was calculated at admission and on day 7 through the calculator in www.mdcalc.com [14, 15]. The Acute Physiology and Chronic Health Evaluation (APACHE), known as a commonly used severity-scoring system, was measured for all patients at admission [16].

Blood sampling was obtained from the vein for measurement of laboratory parameters included CBC-diff, via a standardized automatic cell counter, CRP using the ELISA method, ESR was measured using an automated erythrocyte sedimentation rate analyzer, serum levels of IL6, TNF-ɑ, and IFN-γ using an enzyme-linked immune-sorbent assay, and the blood concentration of vitamin A, vitamin B9, vitamin B 12, vitamin C, vitamin E, and vitamin E by high-performance liquid chromatography (HPLC).

### Sample size

No previous study has been performed on the effect of multivitamins on COVID-19, therefore, the number of samples was calculated based on the following formula. This study included at least 27 people to estimate the mortality rate of 70% in the control group with COVID-19 disease admitted to the intensive care unit, versus of 30% mortality rate in the intervention group. Therefore, the researchers planned to include 30 people in each group in the present study.

$$ n=\frac{{\left({Z}_{1-\alpha /2}+{Z}_{1-\beta}\right)}^2\times \left[{p}_1\times \left(1-{p}_1\right)+{p}_2\times \left(1-{p}_2\right)\right]}{{\left({p}_1-{p}_2\right)}^2} $$

$$ 27=\frac{{\left(1.96+1.28\right)}^2\times \left[0.7\times \left(1-0.7\right)+0.3\times \left(1-0.3\right)\right]}{{\left(0.7-0.3\right)}^2} $$

$$ \alpha =0.05 $$

$$ \beta =0.10 $$

$$ {Z}_{1-\alpha /2}=1.96 $$

### Statistical analysis

Data analyses were conducted via the IBM SPSS Statistics software (version 17). The continuous variable data were presented as mean and standard deviation (SD) or median and interquartile range (IQR) for normally distributed variables, and non-normally distributed variables respectively. Continuous data with a normal distribution were compared using the independent *t* test, and data without a normal distribution were compared using the Mann–Whitney *U* test between the study groups. Categorical variables were shown as frequency (percentage) and compared using a *χ*^2^ test. The absolute effect size for intervention was calculated using Cohen d or Cliff delta formula (for continuous outcomes) and adjusted odds ratio (95% CI) or phi coefficient (for categorical outcomes). *P* value was considered significant when *p* < 0.05.

## Results

### Compare the variables between groups at baseline

**Fig. 1:**
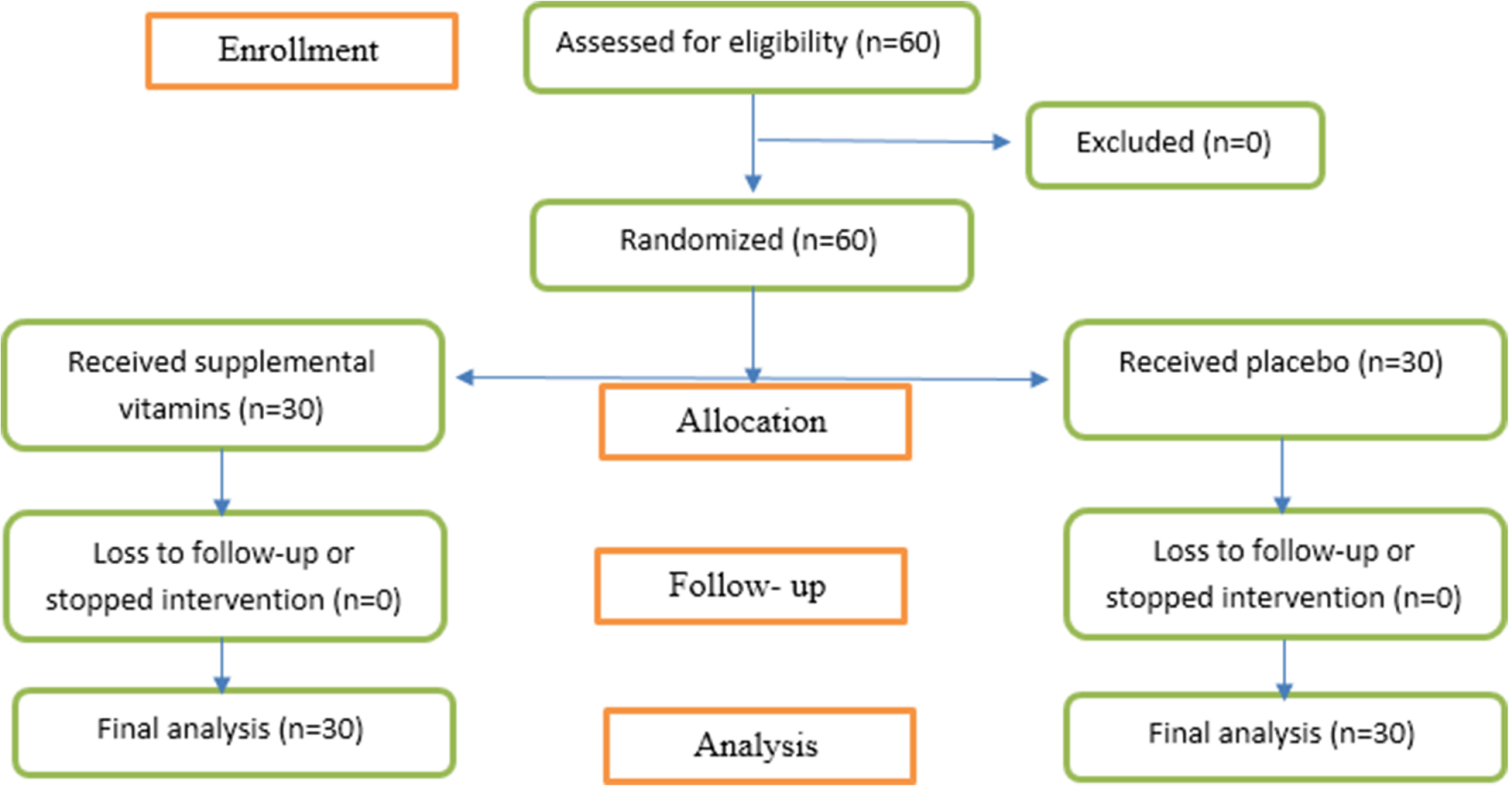
Flow diagram of the study

The baseline characteristics of the patients at enrollment by the treatment group were shown in Table 1. Sixty patients (51.6% men) were enrolled in the study and randomized to intervention (*n*=30) or control (*n*=30) group for 7 days (Fig. 1) that median (IQR) of age was 52.00 (9.00) years (Table 1).

**Table 1:** Baseline characteristics of the patients at enrollment

Variables	Total (***n***=60)	Groups		***P*** value
		Supplementation (***n***=30)	Placebo (***n***=30)	
**Age (year)**	52.00 (9.00)	51.00 (17.25)	53.00 (7.00)	0.437
**Gender,** ***n*** **(%)**				
Male	31 (51.6)	15 (50)	16 (53.3)	0.796
Female	29 (48.4)	15 (50)	14 (46.7)	
BMI*	26.04 (2.69)	26.24 (2.61)	26.24 (2.91)	0.997
Temperature*	37.35 (0.54)	37.51 (0.52)	37.30 (0.58)	0.213
**Respiratory aids,** ***n*** **(%)**				
Mask with reserve	13 (21.7)	6 (20)	7 (23.3)	0.989
Simple mask	21 (35)	11 (36.7)	10 (33.3)	
Non-invasive ventilation	22 (36.7)	11 (36.7)	11 (36.7)	
Invasive ventilation	4 (6.6)	2 (6.7)	2 (6.7)	
**Previous supplementation with vitamin D3,** ***n*** **(%)**	39 (65)	17 (56.7)	22 (73.3)	0.176
**Underlying disease,** ***n*** **(%)**				
DM	11 (18.3)	7 (23.3)	4 (13.3)	0.176
Asthma	8 (13.3)	4 (13.3)	4 (13.3)	
Thyroid diseases	6 (10)	4 (13.3)	2 (6.7)	
Malignancy	3 (5)	2 (6.7)	1 (3.3)	
DM and HTN	20 (33.3)	10 (33.3)	10 (33.3)	
**Oxygen saturation**				
Without aid	93.50 (2.75)	87.50 (4.00)	86.00 (2.5)	0.929
**WBC count, × 10**^**9**^**/L**	6.50 (1.45)	6.40 (1.40)	6.90 (2.67)	0.198
Neutrophil percentage*	76.59 (9.45)	77.73 (13.28)	78.20 (10.17)	0.871
**Lymphocyte percentage**	15.70 (9.95)	14.70 (9.5)	20.20 (13.45)	0.882
**Hemoglobin**	12.40 (3.60)	13.40 (4.02)	12.10 (1.92)	0.717
**FBS**	122.50 (63.25)	123.00 (54.75)	107.00 (61.25)	0.888
**HbA1C**	6.65 (1.07)	6.80 (1.02)	6.10 (1.00)	0.436
ESR*	70.78 (29.24)	76.20 (28.85)	67.70 (26.07)	0.236
CRP*	102.66 (55.97)	98.10 (59.54)	105.83 (37.51)	0.550
**IL6**	213.35 (202.30)	214.60 (210.15)	197.95 (167.35)	0.712
**TNF-ɑ**	213.15 (210.58)	256.75 (225.58)	194.75 (176.52)	0.564
**IFN-γ**	84.65 (123.20)	77.35 (135.18)	147.80 (117.50)	0.882
**Vitamin A**	0.20 (0.20)	0.20 (0.20)	0.20 (0.22)	0.816
Vitamin B9*	6.56 (2.80)	7.90 (3.80)	6.54 (3.10)	0.137
Vitamin B12*	530.21 (308.94)	480.34 (292.71)	521.25 (324.67)	0.610
**Vitamin C**	0.20 (0.10)	0.20 (0.20)	0.10 (0.10)	0.938
**Vitamin D**	22.00 (10.42)	22.00 (9.07)	22.00 (12.35)	0.254
Vitamin E*	10.99 (3.22)	11.30 (3.60)	11.01 (2.53)	0.723
**APACHE score**	20.50 (7.00)	20.00 (7.25)	22.50 (7.25)	0.188
**SOFA score**	7.00 (2.75)	7.00 (2.25)	7.00 (3.00)	0.566

*BMI*, body mass index; *DM*, diabetes mellitus; *HTN*, hypertension; *WBC*, white blood cell; *FBS*, fasting blood sugar; *HbA1c*, hemoglobin A1c; *ESR*, erythrocyte sedimentation rate; *CRP*, C-reactive protein; *IL6*, interleukin-6; *TNF-ɑ*, tumor necrosis factor-ɑ; *IFN-γ*, interferon gamma

*Normally distributed variables

Both age and gender distributions were comparable between the study groups. There was no significant difference in BMI and body temperature between the groups. Oxygen saturation with respiratory aid was significantly lower in the intervention group than in the control group (*p* < 0.001). The usage rate of invasive and noninvasive respiratory aids was almost equal between the study groups at baseline (*p*=0.989). There was no significant difference in the frequency of underlying diseases between the study groups (*p*=0.176) (Table 1).

The baseline laboratory measurements, including CBC count, differential count, ESR, CRP, IL-6, TNF-a, IFN-γ, and levels of vitamins, showed no significant differences between the two groups. The baseline assessments of APACHE (*p*=0.188) and SOFA (*p*=0.566) scores were comparable between the groups (Table 1).

### Compare the variables between groups after the intervention

The follow-up data of inflammatory markers, SOFA score, and total effect size of intervention for each variable were shown in Table 2. There was no significant difference between groups in the change from baseline in WBC and neutrophil count after 7 days (*p* = 0.209 and *p* = 0.494, respectively). There were significant differences in changes in ESR (ES: − 0.98, *P* < 0.001), CRP (ES: − 0.91, *p* = 0.001), IL-6 (ES: − 0.81, *p* = 0.003), and TNF-ɑ (ES: − 0.64, *p* = 0.001) after 7 days from baseline, between the two groups. We did not find significant differences in changes of IFN-γ after supplementation between two groups (ES: − 0.51, *p* = 0.089).

**Table 2:** Follow-up data of inflammatory outcomes and Sofa score between study groups

Variables (at day 7)	Group		Effect size**	***P*** values of MD between groups
	Supplementation	Placebo		
**Sofa score**	3.00 (1.25)	5.50 (5.75)	− 1.1 (L)	< 0.001
Median (IQR) of change from baseline	− 3.50 (1.50)	− 2.00 (4.00)		
*P* value	< 0.001	0.478		
**WBC count, × 10**^**9**^**/L**	9.30 (4.82)	11.05 (3.55)	0.3 (S)	0.209
Median (IQR) of change from baseline	2.65 (4.10)	3.50 (5.32)		
*P* value	0.001	< 0.001		
**Neutrophil count, × 10**^**9**^/L*	71.32 (17.78)	74.26 (11.15)	− 0.2 (S)	0.494
Mean change from baseline	− 6.40 (17.47)	− 3.97 (8.29)		
*P* value	0.054	0.014		
ESR*	43. 83 (27.07)	68.30 (35.89)	− 1.0 (L)	< 0.001
Mean change from baseline	− 32.36 (19.68)	0.60 (43.24)		
*P* value	< 0.001	0.782		
CRP*	34.00 (37.16)	93.07 (56.08)	− 0.9 (L)	0.001
Mean change from baseline	− 64.10 (55.76)	− 12.76 (57.50)		
*P* value	< 0.001	0.234		
**IL6**	86.80 (102.50)	130.20 (254.25)	− 0.8 (M)	0.003
Median (IQR) of change from baseline	− 86.70 (92.10)	− 22.75 (105.62)		
*P* value	< 0.001	0.045		
**TNF-ɑ**	99.25 (80.72)	144.95 (328.18)	− 0.6 (M)	0.001
Median (IQR) of change from baseline	− 96.00 (158.42)	− 20.70 (100.70)		
*P* value	< 0.001	0.043		
**IFN-γ**	38.00 (52.82)	74.30 (114.60)	− 0.5 (S)	0.089
Median (IQR) of change from baseline	− 42.35 (77.42)	− 29.95 (102.75)		
*P* value	< 0.001	0.016		

*WBC*, white blood cell; *ESR*, erythrocyte sedimentation rate; *CRP*, C-reactive protein; *IL6*, interleukin-6; *TNF-ɑ*, tumor necrosis factor-ɑ; *IFN-γ*, interferon gamma; *S*, small: 0.2; *M,* moderate: 0.5; *L*, large: 0.8

*Normally distributed variables

**The effect sizes were calculated by Cohen’s *d* formula or Cliff’s delta statistic

The significant increase from baseline in mean of serum levels of all vitamins including vitamin A, vitamin B9, vitamin B12, vitamin C, vitamin D, and vitamin E were seen after 7 days of intervention. The effect size of treatment was significant between the study groups (ESs were 1.93, 1.01, 2.35, 2.26, 1.64, and 2.55, respectively; *p* < 0.001 for all comparisons) (Table 3).

**Table 3:** Follow-up data of serum levels of vitamins by study groups

Variables (at day 7)	Group		Effect size**	***P*** values of changes between groups
	Supplementation	Placebo		
**Vitamin A**	0.35 (0.50)	0.10 (0.20)	1.9 (L)	< 0.001
Median (IQR) of change from baseline	0.20 (0.20)	0.00 (0.10)		
*P* value	< 0.001	0.035		
Vitamin B9*	10.49 (3.87)	5.64 (2.34)	1.0 (L)	< 0.001
Mean change from baseline	2.60 (4.16)	− 0.91 (2.62)		
*P* value	0.002	0.068		
Vitamin B12*	889.00 (389.16)	414.24 (301.33)	2.4 (L)	< 0.001
Mean change from baseline	410.02 (263.55)	− 107.00 (165.61)		
*P* value	< 0.001	0.001		
**Vitamin C**	0.40 (0.10)	0.10 (0.10)	2.3 (L)	< 0.001
Median (IQR) of change from baseline	0.20 (0.20)	0.00 (0.10)		
*P* value	< 0.001	0.011		
**Vitamin D**	29.75 (15.00)	19.55 (8.37)	1.6 (L)	< 0.001
Median (IQR) of change from baseline	9.50 (10.85)	− 6.40 (8.52)		
*P* value	< 0.001	< 0.001		
Vitamin E*	13.86 (3.78)	8.30 (2.17)	2.6 (L)	< 0.001
Median (IQR) of change from baseline	2.56 (1.57)	− 2.71 (2.47)		
*P* value	< 0.001	< 0.001		

*S*, small: 0.2; *M*, moderate: 0.5; *L*, large: 0.8

*Normally distributed variables

**The effect sizes were calculated by Cohen’s *d* formula or Cliff’s delta statistic

Table 4 shows the rate of mortality and prolonged hospitalization in the study groups. Mortality was 0% in the supplemental vitamin group and 13.3% in the placebo group, which this difference was not statistically significant (*p* = 0.112). The prolonged hospitalization rate of over 7 days was 13% and 53% in patients assigned to supplemental vitamins and placebo, respectively (*p* = 0.001). In the next step, the effect size was evaluated for the significant difference in the prolonged hospitalization rate between the study groups. The Phi coefficient of 0.424 was achieved, which indicated an inverse, relatively strong association between receiving supplemental vitamins and prolonged hospitalization [17]. Measuring the effect size with multivariate logistic regression analysis showed that the odds of prolonged hospitalization in patients who received supplemental vitamins was 40% of the odds for the placebo group; however, this OR was not statistically significant after adjusting for potential confounders (OR: 0.402, 95% CI 0.086–1.883; *p* = 0.247). We did not detect any complications and side effects in the present study.

**Table 4:** Binary outcomes between study groups

Outcomes	Groups		***P*** value	Effect size (if chi square of fisher exact test is significant)	
	Supplementation (***N***=30)	Placebo (***N***=30)			
**Mortality rate,** ***n*** **(%)**	0 (0)	4 (13.3%)	0.112	-	
**Prolonged hospitalization > 7 days,** ***n*** **(%)**	4 (13%)	16 (53%)	0.001	Phi = 0.424	Unadjusted OR: 0.135 (95% CI 0.038–0.481; *p*=0.002) Adjusted OR*: 0.402 (95% CI 0.086–1.883; *p*=0.247)

*Adjusted for age, gender, and O_2_ saturation

## Discussion

### Generalizability

In this study, supplementation with vitamins included A (25,000 IU daily), D (600,000 IU once during the study), E (300 IU twice daily), C (four times daily), and B complex (daily) for 7 days significantly decreased the serum levels of inflammatory markers and severity of the disease. Moreover, the supplementation reduced the prolonged hospitalization rate, although the likelihood of this effect was not significant after adjusting for confounders and had no beneficial effect on the mortality rate in patients with COVID-19 admitted in ICU.

We determined the dose of vitamins for compensating the probable deficiencies or increased demand due to COVID-19, which was shown compared to the control group after 7 days of supplementation.

To our knowledge, few clinical trials are so far available in the literature that evaluated the effect of vitamin supplements on COVID-19. A pilot trial study by Castillo et al. assessed the beneficial role of high dose calcifediol (25-hydroxyvitamin D3) in patients admitted with COVID-19 and reported a significant decrease in the rate of need for ICU admission and the severity of these patients. The main limitations of Castillo et al.’s study were designing as a single-blinded study and ignoring the assessment of BMI as a potential confounder for the severity of the COVID-19 disease [18]. Our study was also a single-blind study but evaluated the baseline BMI for the patients in study groups and BMI was not significantly different between the study groups.

A quasi-experimental study by Annweiler et al. has figured out that patients regularly supplemented with vitamin D before COVID-19 contracting were less likely to develop the severe form of the disease with respect to those supplemented after COVID-19 contracting. This study mentioned that the time for vitamin D supplementation, before or after COVID-19 contracting, is a challenging issue. Moreover, because of the nonrandom sampling in this study, the risk of selection and confounding biases is probable. In the end, this study has recommended to replacement the vitamin D in all people [12].

Calcitriol is an active form of vitamin D3 and vitamin E that have improved the innate and adaptive immune functions [19–21]. Contrary to Annweiler et al.’s results, our study had a random assignment, tested several types of vitamins, and found a significant improvement in the severity of disease determined by reduced the SOFA score, in patients who received vitamins, compared to the control group. The previous history of supplementation with vitamins and the baseline serum levels of vitamins did not differ between the study groups in our study; therefore, these items did not confound the evaluation of treatment effect with vitamins.

The effect size of treatment for SOFA score was − 1.10, which indicates a considerable strength according to statistical evidence [17]. This result was also in line with the last shreds of evidence on the association between micronutrient deficiencies and increased severity of COVID-19 infection [18, 22, 23].

As mentioned earlier, one of the main factors influencing the severity of the COVID-19 disease is impaired immune response and subsequent over-exuberant inflammatory response to infection [3–6]. Clinical studies have illustrated the impact of supplementation with various vitamins on different stages of immune responses; in this way, vitamin A could improve the function of innate immune cells such as neutrophils, NK cells, and macrophages, and antibody-mediated responses to infection [19, 24]. In addition, vitamin B6 and vitamin B12 have had a beneficial impact on the adaptive immune response by helping increase and maturing lymphocyte cells. Also, high doses of vitamin B6 have been found useful in modulating the immune responses of critically ill patients [25, 26].

Regarding vitamin C supplementation, it provokes phagocyte and T-lymphocyte cells and protects them from oxidative stress [19, 27]. Moreover, high doses of vitamin C have been effective for speeding the recovery of critically ill patients in ICU [28]. Given the synergistic effect of vitamins in strengthening the immune system function, as well as the critical and ambiguous evidence on the behavior of COVID-19, for early recovery in critically ill patients and decrease the mortality rate, we try evaluating the effect of a combination of vitamins in patients admitted in ICU. Finally, we found that vitamins are useful in reducing serum inflammatory markers with respect to the control group, but further study of the issue is still required.

Evidence have shown vitamins A, B, C, and E could be effective on different components of innate immunity and prevent the cell tissue injury in the defense process against infections. Deficiencies of them may disturb the function of natural killer cells. However, the specific effects of micronutrients on neutrophil functions are still not clear [28, 29].

### Strength points

Our study was the first RCT to examine whether simultaneous usage of supplements included different vitamins has beneficial effects on clinical and laboratory outcomes of patients with COVID-19 admitted to ICU. Other strength points were the homogenous group of patients and the complete follow-up for the intention-to-treat analysis of the main endpoints. However, this study could not show the effect of any type of vitamins separately, we have expressed our applicable results of the synergistic effect of simultaneous usage of all vitamins that are recommended in numerous studies especially in the critical situation of COVID-19 all over the world. This study recommended further studies that result in anti-coronavirus multivitamin production.

### Study limitations

At the beginning of the COVID-19 critical situation in the hospital, we faced some unavoidable limitations for the study that some of them are mentioned here. However, we achieved our sample size which could not enroll more patients because of tight inclusion criteria and financial issues. Furthermore, in the situations that we are worried about the rate of mortality in patients that were admitted in ICU, and we used no antivirus and immunomodulatory drugs, the simultaneous supplementation with different vitamins was guessed the best way to inhibition of mortality and hospitalize duration for restrained COVID-19. Therefore, we could not ignore any vitamins that considered may be effective on the rate of COVID-19 mortality.

In addition, we could not use placebo because of the critical situation of ICU admitted patients and run a single-blind study. Also, we included elderly patients hospitalized in ICU of a single-center who might be unrepresentative of the population. We could not evaluate serum levels of other micronutrients, dietary assessments, exact weight, exact height, after supplementation chest CT and qRT-PCR Ct, because of the limited finance, time, and hospital facilities. In this way, we recommend further studies to perform without the limitations to get a better result on the mortality rate.

### Changes that occurred to the original protocol

We could not achieve data of CT scans for all patients because of our time and hospital facility limitations.

### Conclusion

Overall, in ICU-admitted patients with COVID-19, supplementation with vitamins A, B, C, D, and E was associated with less severe COVID-19 presentation and reduced serum levels of inflammatory markers. The rate of prolonged hospitalization was lower in patients who received the supplements, though the likelihood of prolonged hospitalization in the supplemental vitamin group was not significant after considering confounders.

## Data Availability

Yes

## Abbreviations

ICU: Intensive-care unit
O_2_: Oxygen
BMI: Body mass index
COVID-19: Coronavirus 19
ESR: Erythrocyte sedimentation rate
CRP: C-reactive protein
IFN-g: Interferon-gamma
TNF-a: Tumor necrosis factor-alpha
IL-6: Interleukin-6
APACHE score: Acute Physiologic Assessment and Chronic Health Evaluation
CT scan: Computed tomography scan
SOFA: Sequential Organ Failure Assessment
CBC-diff: Complete vlood cell count and differential count
ES: Effect size
